# Resection of Primary Renal Leiomyosarcoma Involving the Inferior Vena Cava (IVC) with IVC Resection and Reconstruction

**DOI:** 10.1155/2022/6037890

**Published:** 2022-06-08

**Authors:** Khaled Alshawwa, Jamal Hijazi, Bashar Jaber, Haitham AlHassan, Thaer Abu-Amer, Riyad Salahaldeen, Omar Abu-Zaydeh, Riad Haddad

**Affiliations:** ^1^Al-Makassed Islamic Charitable Society Hospital, Jerusalem, State of Palestine; ^2^Shaare Zedek Medical Center, Jerusalem, State of Palestine; ^3^Carmel Medical Center, Haifa, State of Palestine

## Abstract

Renal leiomyosarcoma is a rare, aggressive tumor of the smooth muscles of the kidney. In our case, the tumor has special characteristics that made it highly challenging, as it involved major vessels and other adjacent vital structures. The rarity of the tumor type itself and the special challenging features we faced intraoperatively encouraged us to report the case including the management plan for R0 resection. Our patient is a forty-two years old previously healthy female, with vague nonspecific presenting complains, ended up with a major highly advanced surgery necessitating the need for vascular reconstruction of IVC. The surgery was performed by a multidisciplinary team of highly specialized surgeons in related fields. The surgery went well, and the outcome was promising. The patient was followed up for about four months later, with uneventful course.

## 1. Background

Renal leiomyosarcoma (LMS) is a rare and aggressive mesenchymal tumor that usually arises from the smooth muscle cells of the intrarenal blood vessels or the renal pelvis [1]. Renal sarcomas represent 1-2% of malignant kidney tumors in adults; 50% of renal sarcomas is leiomyosarcoma [2].

Leiomyosarcoma has high tendency of local recurrence; hence, complete surgical excision is recommended [1]. The typical presentation is between the 4th and 6th decades of life with a mean age at diagnosis of 58.5 (range 22-85) [1]. The most common presenting sign is an abdominal mass with or without pain and hematuria, similar to renal cell carcinoma [3].

A margin-free resection may be curative, but the resection must involve the tumor en bloc with the affected segment of vena cava and locally involved organs. IVC resection often requires vascular reconstruction, which can be done with prosthetic graft [4].

Literature review showed that reconstruction of IVC and RV is usually not necessary after the resection of IVC in retroperitoneal mass excision [5], but in our case, reconstruction and anastomosis of left renal vein to IVC graft was not avoidable, as right kidney was resected completely with the tumor.

A review of a case series reported 12 patients with 6 primary IVC-tumors (leiomyosarcoma) and 6 secondary IVC-tumors (2 retroperitoneal tumor lesions, 3 renal cell carcinomas 25%, and 1 carcinoma of the adrenal gland). The RO resection rate was 83%. The perioperative morbidity was 33%, whereas the hospital mortality was 8.3% (*n* = 1). Surgical reconstruction of IVC was achieved in each case (100%). The favorable outcome of this case series demonstrates that IVC-associated tumor lesions can be approached if there is an appropriate expertise of the surgical team, a sufficient perioperative management, and an adequate financial background with a reasonable survival rate. The variable prognosis of the various tumor lesions depends on tumor entity, stage, resection status, and individual risk factors [6].

A complete surgical resection is the only proven therapeutic modality that prolongs the survival in patients with leiomyosarcoma of the inferior vena cava (IVC) or invasion of IVC from adjacent leiomyosarcoma of other organ. Reconstruction of the IVC is not always necessary but is often required to facilitate venous drainage of the kidney for the tumors at the pararenal area of the IVC, or—as in our case—when one or both kidneys is at risk [7]. So when (IVC) resection is required, reconstruction is recommended. This is particularly true when the renal vein confluence is resected to preserve venous outflow, including that of the kidney [8].

Histologically, leiomyosarcoma of kidney has to be differentiated from sarcomatoid renal cell carcinoma, leiomyoma, and angiomyolipoma. Immunohistochemically, the tumor cells showed diffuse and strong positivity for smooth muscle actin (SMA), desmin, and vimentin [9].

Primary renal extraskeletal Ewing sarcoma is an another rare renal mesenchymal neoplasm, often metastatic at diagnosis, and with a poor outcome [10].

It is worthy to mention that other rarer renal mesenchymal tumors that can occur in adults include angiosarcoma (less than 5% of angiosarcomas arises in the genitourinary tract), rhabdomyosarcoma, osteosarcoma (there are fewer than 30 reported cases of primary renal osteosarcoma), synovial sarcoma, Ewing sarcoma, angiomyolipoma, epithelioid angiomyolipoma, leiomyoma, hemangioma, lymphangioma, hemangioblastoma, juxtaglomerular cell tumor, and schwannoma.

## 2. Case Report

**Figure 1:**
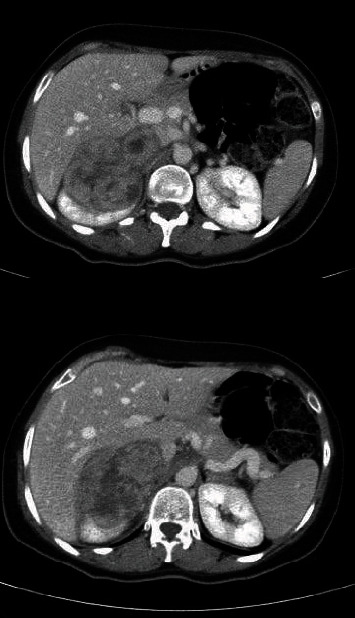
Axial CT views of the lesion, showing the involvement of the right kidney and IVC.

**Figure 2:**
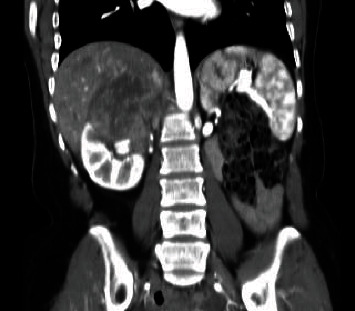
Coronal CT view of the lesion, showing the involvement of the right kidney and IVC.

**Figure 3:**
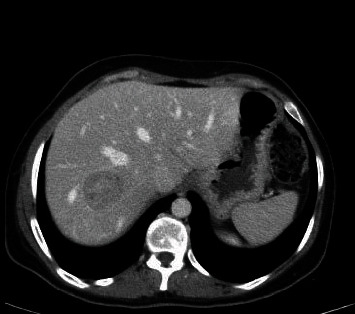
Axial CT view of the lesion, showing the lesion invading the liver parenchyma.

A 42-years-old female patient started to complain from intermittent attacks of dull aching RUQ and epigastric abdominal pain. Physical examination revealed mild RUQ tenderness, without palpable mass. Abdominal ultrasound revealed a heterogeneous lesion at upper pole of right kidney. A computerized tomography (CT) was performed and showed heterogeneous lesion at the upper pole of the right kidney that measures 7.5 × 7 cm, invading adjacent liver parenchyma without clear cleavage (Figures 1–3).

Abdominal MRI was done next and showed a heterogeneously enhanced soft tissue mass in the upper pole of the right kidney measuring 6.8∗9.5 cm with the involvement mainly of segment VII of the liver and a small part of segment VIII of the liver. The mass abutting IVC and severely narrowing it appearing slit-like. However, the lower segment of IVC wall looks involved by the mass.

Ultrasound guided tru-cut biopsy was performed and showed histopathologic evidence of leiomyosarcoma with many mitotic figures.

The case was discussed with oncologists, surgeons, radiologists, and pathologists in MDT meeting, and the decision was to go for surgery intending to achieve R0 resection of the tumor.

Although evidence showed that renal artery embolization before radical nephrectomy for renal masses seems to be a useful tool in surgical management of a large mass and advanced disease, as it induces preoperative infarction and facilitates surgical intervention [11], this was not possible in our patient, as radiological evaluation did not certainly confirm renal versus hepatic origin of the tumor, and there was no single certain feeding vessel.

Surgery was performed through Makuuchi incision. Intraoperatively, a large solid retroperitoneal mass was found, engulfing and invading IVC to the level of the left renal vein and extending upwards to the hepatic veins and compressing the right liver lobe (segment VII).

Right nephrectomy, IVC segment resection (from insertion of the hepatic vein reaching the level of the left renal vein), and complete excision of the tumor were performed.

As there was no previous radiological evidence nor intraoperative grossly detectable prominent collateral venous drainage to IVC, IVC graft implantation was necessary.

**Figure 4:**
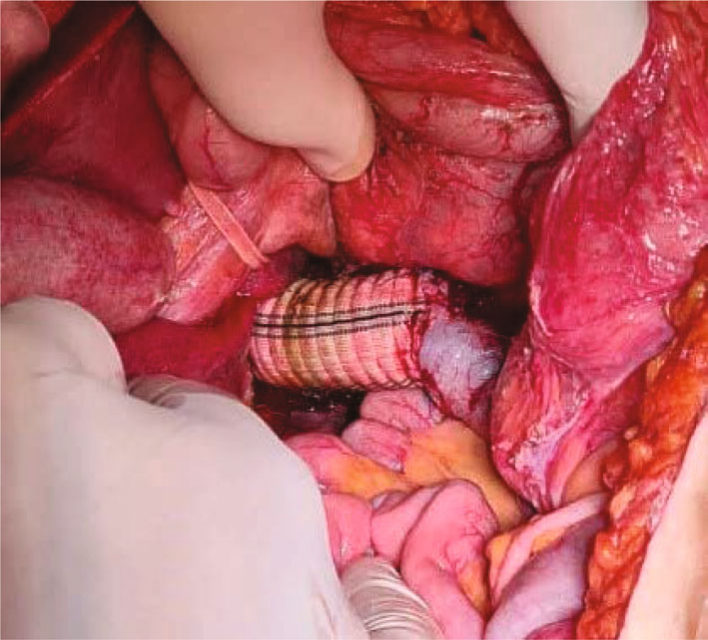
Intraoperative image showing the implanted IVC Dacron graft at the level of the left renal vein.

Reconstruction of the vena cava was performed by the implantation of a 22 mm Dacron graft, anastomosed obliquely to the IVC at the level of the left renal vein. Intraoperative blood loss was about 3 liters, surgery lasted for about seven hours, and there were no intraoperative complications (Figure 4).

Postoperatively, the patient was kept for 48 hours in ICU for close observation then transferred to the general ward. In the following hours, she developed intra-abdominal bleeding with hematoma formation and bloody drain output, managed conservatively with blood products transfusion. She was discharged two weeks later in good general condition.

A week after discharge, she complained of abdominal pain and distention, imaging revealed an accumulation of intraperitoneal fluid, and an ultrasound-guided drain was inserted, draining old blood with successful resolving of her symptoms.

Histopathology report of the specimen showed a 9 cm leiomyosarcoma predominately involving the kidney (upper half) and pushes into the renal sinus and ipsilateral adrenal gland, with a negative ureteric margin. Separate vascular wall pieces with chronic inflammation and focal mural thrombosis were negative for tumor. The tumor seems to be limited by a thin fibrous capsule superiorly. By immunohistochemistry, the tumor cells are positive for SMA and desmin.

A CT scan follow up at two months showed only postoperative changes, with no evidence of metastatic disease or local recurrence. Patient was followed up closely for four months after surgery, and her condition showed an uneventful course. She was sent to an oncological center and started on a radiotherapy course.

## 3. Conclusion

Invasive leiomyosarcoma of the kidney represent a technical challenge. Careful preoperative investigation and an experienced dedicated team consisting of vascular, urologic, and—in our case—hepatobiliary surgeons are usually needed for a safe and successful R0 resection despite extensive tumor involvement.

## Data Availability

Other clinical data or figures supporting diagnosis or management are available from the authors upon request.
